# Policy search with rare significant events: Choosing the right partner to cooperate with

**DOI:** 10.1371/journal.pone.0266841

**Published:** 2022-04-26

**Authors:** Paul Ecoffet, Nicolas Fontbonne, Jean-Baptiste André, Nicolas Bredeche

**Affiliations:** 1 Sorbonne Université, CNRS, Institut des Systèmes Intelligents et de Robotique, ISIR, Paris, France; 2 Institut Jean Nicod, Département d’Études Cognitives, École Normale Supérieure, Paris, France; Kyushu Daigaku, JAPAN

## Abstract

This paper focuses on a class of reinforcement learning problems where significant events are rare and limited to a single positive reward per episode. A typical example is that of an agent who has to choose a partner to cooperate with, while a large number of partners are simply *not* interested in cooperating, regardless of what the agent has to offer. We address this problem in a continuous state and action space with two different kinds of search methods: a gradient policy search method and a direct policy search method using an evolution strategy. We show that when significant events are rare, gradient information is also scarce, making it difficult for policy gradient search methods to find an optimal policy, with or without a deep neural architecture. On the other hand, we show that direct policy search methods are invariant to the rarity of significant events, which is yet another confirmation of the unique role evolutionary algorithms has to play as a reinforcement learning method.

## 1 Introduction

We consider a particular class of reinforcement learning problems where only rare events can result in non-zero rewards *and* when the agent can experience at most one positive reward in a limited time. This problem is closely related to the problem of learning with **rare significant events** in reinforcement learning [1–3], where rare events can significantly affect performance (e.g. in network and communication systems or control problems where failure can be catastrophic). In this paper, we consider that while significant events occur independently of the agent’s actions, the agent’s policy determines if a positive reward should be obtained when such an event occurs. Significant events are thus defined as unique opportunities to obtain a positive reward and stop the game. Each opportunity can either be seized for an immediate reward or ignored if the agent hopes to get a better reward in the future.

We address this problem in the context of an **independent, on-line and on-policy episodic learning task** with continuous state and action spaces. The practical application addressed in this paper is that of an agent learning to choose a partner for a task that requires cooperation (e.g., predators hunting a large prey or individuals selecting a lifelong mate). The agent can choose to cooperate or not with a potential partner, based on the effort this partner is willing to invest in the cooperation. At the same time, the agent must invest enough so that its partner also accepts to cooperate. In this setup, the agent may face partners willing to invest various amount of energy in cooperation (i.e., a possibly significant event), or even refuse to cooperate whatever the agent is ready to invest (i.e. a non-significant event).

Results from theoretical biology [4–7] have shown that cooperation with partner choice is optimal only under certain conditions. First, the number of cooperation opportunities must be large enough that an agent can refuse to cooperate with a potential partner and still have the opportunity to meet a more interesting partner. Second, if an agent and its partner both decide to cooperate, the actual duration of this cooperation must be long enough to make cooperation with an uninteresting partner significantly costly (which is the case when there can be only one single successful cooperation event). Under these conditions, the optimal strategy for an agent is to be very demanding in choosing its partner.

The question raised in this paper is whether reinforcement learning algorithms actually succeed in learning an optimal strategy when the necessary conditions are met. We are particularly interested in how the rarity of significant events influences convergence speed and performance of policy learning. Indeed, it is not clear how gradient-based policy search method can deal with a possibly large number of non-significant events that provide zero-reward.

We use two state-of-the-art methods for on-policy reinforcement learning with continuous state and action spaces: (1) a deep learning method (PPO [8]) for gradient policy search and (2) an evolutionary method (CMAES [9]) for direct policy search. While both methods provide similar results when the agent is always presented with significant events, policy search methods are not equals when such events become rarer. While the direct policy method is oblivious to rarity of significant events, the gradient policy search method suffers significantly from rarity.

The paper is structured as follows: the reinforcement learning problem with significant rare events and single reward per episode is formalized, and the partner choice learning problem is presented as a variation of a continuous prisoner’s dilemma. Algorithms and results are then presented, and learned policies are analysed and compared.

## 2 Methods

In this Section, we start by using the framework of reinforcement learning to formalize the problem of learning with rare significant events (Subsection 2.1). Then, we detail the payoff function used to compute the agent’s instantaneous reward when cooperation actually occurs (Subsection 2.2), which value depends on (1) how much agents are ready to invest and (2) whether both agents decide to accept (or not) to actually cooperate depending on what their respective partner’s investment. Finally, we describe how partner choice is actually implemented from an agent’s viewpoint (Subsection 2.3), which implies using both (1) an investment module used by an agent to provide the cost it is willing to pay for cooperation, and (2) a choice module used by an agent to decide to pursue cooperation or not depending on the cost its partner is ready to invest in cooperation.

For convenience, all important notations introduced in this Section and used afterwards are summarized in the Annex at the end of the main text.

### 2.1 Learning with rare significant events

Formally, we consider an independent learner *x*_•_, called the *focal agent*, which is placed in an aspatial environment. At each time step, *x*_•_ is presented with either a *cooperative partner* $x_{i}^{+}\in X^{+}$ or a *non-cooperative partner* $x_{j}^{-}\in X^{-}$. *X*^+^ (resp. *X*^−^) is the finite set of all cooperative (resp. non-cooperative) agents, with both *i* and j∈N and *i* > 0, *j* ≥ 0. When presented with a non-cooperative partner $x_{j}^{-}$, the focal agent’s reward will always be zero. When presented with a cooperative partner $x_{i}^{+}$, the focal agent’s reward will depend on its own action and that of its partner. (see Section 2.2 for details).

Our objective is to endow the focal agent *x*_•_ with the ability to learn how to best cooperate, which implies to negotiate with its potential partners and decide whether cooperation is worth investing energy in, or not (see Section 2.3 for details). The focal agent faces an individual learning problem as it must optimize its own gain over time in a competitive setup, whether its partners are also learning agents or not. For cooperation to occur between the focal agent and a partner, the partner must willing to cooperate (ie. be one of $x_{i}^{+}$) and both the focal agent *and* the cooperative partner must estimate that one’s own energy invested in cooperation is worth the benefits.

We use the standard reinforcement learning framework proposed by Sutton et al. [10] to formalize the learning task from the focal agent’s viewpoint, which is essentially a single agent reinforcement learning problem.

The focal agent *x*_•_ interacts with the environment in a discrete time manner. At each time step *t* = 0, 1, 2, …, *x*_•_ is in a state s∈R which describes its current partner’s investment value, and plays a continuous value a∈R which represents its decision to cooperate (*a* > 0) or not (*a* <= 0).

Let *π*_*θ*_ be the parametrised policy of the focal agent, with $\theta \in R^{n}$. The learning task is to search for θ*, such as:

$$\begin{array}{c} \theta^{*}=\underset{\theta }{argmax}J\left(\theta \right) \end{array}$$ (1)

With *J* the global function to be optimized, defined as:

$$\begin{array}{c} J\left(\theta \right)=E\sum_{t}r_{t} \end{array}$$ (2)

with reward *r*_*t*_ at time *t*. Rewards are defined such that r∈R and depends on the current state *s* and action *a*, and are produced according to the probability generator defined as follow:

$$\begin{array}{c} r\left(s,a\right)=\{\begin{array}{cc} payoff(s,a) & \text{with}\;\text{probability}\;p\\ 0 & \text{otherwise}. \end{array} \end{array}$$ (3)

The probability *p* ∈ [0, 1] determines the probability to encounter a cooperative agent (i.e. one of $x_{i}^{+}$). The value of *p* depends on the setup, and determines how *rare* significant events occur when *p* < 1.0. A probability of *p* = 1.0 means the focal agent *x*_•_ encounters a cooperative partner at each time step *t*, with a possible positive reward (if cooperation is accepted by both agents) that depends on the *payoff* function. Non-zero rewards become rarer (but still possible) as *p* → 0. Note that *payoff*(*s*, *a*) is non-zero *only* if both the focal agent *and* its cooperative partner accept to cooperate. Cf. Section 2.3 for details on the negotiation process.

The problem presented here is very similar to that of Rare Significant Events as formulated by Frank et al. [2]. However, our problem differs on two aspects. Firstly, we consider on-line on-policy search of a parametrised policy, where the frequency of significant events cannot be controlled. Secondly, and even more importantly, a learning episode stops right after the focal agent and one cooperative agent have reached a consensus to cooperate. If no cooperation is triggered, an episode stops after a maximum number of iterations *T*, defined as:

$$\begin{array}{c} T=\frac{100}{p}\;\text{time}\;\text{steps} \end{array}$$ (4)

It results that the expected number of meetings *M* is held constant independently from the value of *p* (i.e. E(M)=100). It is therefore possible to obtain episodes of different lengths but with the same number of significant events.

The situation that is modelled here corresponds to many collective tasks observed in nature [11–13], where each agent has to balance between looking for partners and cooperating with the current partner, the latter possibly taking significant time. As a matter of fact, it has been shown elsewhere [4–7, 14] that optimal partner choice strategies can be reached only when the cost of cooperation is large (ie. the duration of cooperation is long with regards to looking for cooperative partners).

### 2.2 Partner choice and payoff function

Whenever the focal agent *x*_•_ and a cooperative partner $x_{i}^{+}$ interact together, they play a variation of a continuous Prisoner’s Dilemma. Cooperation actually takes place if *both* agents deem it worthwhile. The two-step procedure for partner choice is the following:

1. each agent simultaneously announce the *investment* they are willing to pay to cooperate;
2. each agent then *chooses* to continue the cooperation based on the investment announced by its partner and its own.

To simplify notations, we use *x*_•_ and $x_{i}^{+}$ to represent both the agents and the investment values they play, i.e. *x*_•_ (resp. $x_{i}^{+}$) plays *x*_•_ (resp. $x_{i}^{+}$). The gain received by the focal agent *x*_•_ is defined as:

$$\begin{array}{c} P\left(x_{•},x_{i}^{+}\right)=a\times x_{•}+b\times x_{i}^{+}-\frac{1}{2}x_{•}^{2} \end{array}$$ (5)

With *a*, *b* ≥ 0 and *a* + *b* > 0. This payoff function combines both a prisoner’s dilemma and a public good game, and was first introduced in our previous work [7] (a broad introduction to evolutionary game theory can be found elsewhere [15–18]). Two different equilibria can be reached for *x*_•_:

- *x*_*d*_ = *a*. This is a sub-optimal equilibrium, which corresponds to an agent cheating, a typical outcome in the prisoner’s dilemma where an agent maximizes its own gain, but also minimizes its exposure to defection. This ensure the best payoff for the agent if it is unable to distinguish a cheater from a cooperator.
- *x*_*c*_ = *a* + *b*. This is the optimal equilibrium, where both agents cooperate to maximize their long-term gain.

The public good game is included in the payoff function to help distinguish between agents that are simply ignoring the cooperation game (*x*_•_ = 0), from those who takes part in it, even if they defect (*x*_•_ ≥ *x*_*d*_).

The focal agent can get the optimal payoff if it plays *x*_•_ = *x*_*c*_ *and* its partner plays $x_{i}^{+}\geq x_{c}$, which can occur if particular conditions are met when partner choice is enabled. Partner choice can lead to optimal individual gain whenever a successful cooperation removes the possibility for further gain with other partners. In other words: the focal agent can meet with any number of possible partners but will take the gain of the first and single mutually accepted cooperation offer.

It can be noted that *x*_*d*_ and *x*_*c*_ are actually Nash equilibria when all agents are learning. The difference between the two equilibria makes it possible to easily capture the cost paid when agents are *not* cooperating. The interested reader is referred to the concept of Price of Anarchy [19] that is paid by agents that cannot agree to cooperate even when it is in their best interests (see also the work in [20] for a similar concept). A comprehensive analysis of the evolutionary dynamics with and without partner choice using this payoff function can be found in our previous work [7].

In this paper, we set *a* = 5 and *b* = 5, therefore *x*_*d*_ = 5 and *x*_*c*_ = 10. The maximum payoff the agent can obtain is to invest *x*_•_ = *x*_*c*_ with its partner investing equally $x_{i}^{+}=x_{c}$. In this context, $P(x_{•},x_{i}^{+})=50$. The focal agent’s investment is bounded as 0.0 ≤ *x*_•_ ≤ 15.0. This is similar for $x_{i}^{+}$.

$P(x_{•},x_{i}^{+})$ and *payoff*(*s*, *a*) (introduced in Eq 3) differs as the *P* function relates to the game theoretical setting while the *payoff* function relates to the reinforcement learning problem. On the one hand, the *payoff* function computes the focal individual’s reward whether *or not* cooperation was initiated. On the other hand, *P* computes the focal individual’s gain that results from a cooperation game between two agents that *accepted* to cooperate. However, both functions are linked. From a notational standpoint, *s* represents the investment value of the focal individual *x*_•_, and *a* represents the decision to cooperate and depends on both *s* and that of its partner $s_{i}^{+}$ (which is implicit). The return value of *payoff*(*s*, *a*) depends on whether cooperation was initiated or not. If both agents decided to cooperate, then the focal agent’s payoff is $payoff\left(s,a\right)=P\left(x_{•},x_{i}^{+}\right)$, with $P(x_{•},x_{i}^{+})\leq 50$ in this case. If cooperation fails, the focal agent’s payoff is *payoff*(*s*, *a*) = 0 (which is obtained without having to compute *P*). The *payoff* function in Eq 3 can be written as follow, with updated notations and assuming *a*_•_ > 0 (resp. $a_{i}^{+}>0$) means the focal agent (resp. partner) is willing to cooperate:

$$\begin{array}{c} payoff\left(s_{•},a_{•}\right)=\{\begin{array}{cc} P(x_{•},x_{i}^{+}) & \text{if}\;a_{•}>0\;\text{and}\;a_{i}^{+}>0\\ 0 & \text{otherwise}. \end{array} \end{array}$$ (6)

### 2.3 Behavioural strategies

For each interaction, the focal agent’s investment value *x*_•_ ∈ [0, 15] is computed, and when the investment value of its partner is known, its decision to cooperate $a_{•}\in R$ is computed to determine if cooperation should be pursued or not. Each value is provided by a dedicated decision module:

- the **investment module** which provides the cost *x*_•_ that the focal agent is willing to invest to cooperate. This module takes no input as it is endogenous to the agent (i.e. the proposed cost *x*_•_ is fixed throughout an episode);
- the **choice module** takes both the focal agent’s own investment value (*x*_•_) and that of its partner ($x_{i}^{+}$ or $x_{j}^{-}$), and computes *a*_•_, which is used to determine if cooperation is an interesting choice (*a*_•_ > 0) or not (*a*_•_ ≤ 0). The choice module is essentially a function *f*_*choice*_(*x*_•_, *x*_*partner*_) → *a*_•_ with *x*_*partner*_ ∈ *X*^+^ ∪ *X*^−^. The parameters of the function are learned, and the decision to cooperate is computed (as the decision to cooperate is conditioned by the partner’s investment).

With respect to the focal individual, Section 3 describes how the investment and choice modules are defined and how learning is performed depending on the learning algorithm used.

Cooperative partners $x_{i}^{+}$ and non-cooperative partners $x_{j}^{-}$ also use similar decision modules, providing investment and choice values. However, all use deterministic fixed strategies, which may differ from one partner to another. Firstly, non-cooperative partners $x_{j}^{-}$ all follow the same strategy. Both the investment value $x_{j}^{-}$ and the decision to cooperate $a_{j}^{-}$ are always 0, ∀*j*.

Secondly, cooperative partners $x_{i}^{+}$ each follows a stereotypical cooperative strategy depending on the value *i*. Each cooperating partner invests a fixed value $x_{i}^{+}\in \left[0,15\right]$ defined as:

$$\begin{array}{c} x_{i}^{+}=\frac{i-1}{i_{max}}\times 15,\;i\in \left\{1,\ldots ,i_{max}\right\} \end{array}$$ (7)

Each cooperative partner then accepts to cooperate if the focal agent’s investment value *x*_•_ is greater or equal to their investment, which is written as follow:

$$\begin{array}{c} a_{i}^{+}=\{\begin{array}{cc} 1 & \text{if}\;x_{•}\geq x_{i}^{+}\\ -1 & \text{otherwise}. \end{array} \end{array}$$ (8)

In the following, there are *i*_*max*_ = 31 cooperating partners ($x_{i}^{+}\in X^{+},i\in \left\{1,\ldots ,31\right\}$). Following Eq 8, this means cooperating partner $x_{1}^{+}$ (resp. $x_{2}^{+}$, …, $x_{31}^{+}$) plays 0 (resp. 0.5, …, 15).

## 3 Parameter settings and algorithms

We use two reinforcement learning algorithms: a gradient policy search algorithm (PPO) and a direct policy search algorithm (CMAES). Both algorithms are used to learn the parameters of the focal agent’s decision modules.

For both algorithms, the performance of a policy (i.e. the *return* or the *fitness*, depending on the vocabulary used) during one episode is computed as the sum of rewards during the episode (cf. Section 2.1), which is either zero, or the value of the unique non-zero reward obtained before the episode ends.

### 3.1 Proximal policy optimization

The deep reinforcement learning Proximal Policy Optimisation (PPO) [8] is a variation of the Policy Gradient algorithm [10]. Policy gradient algorithms maximize the global performance by updating the parameters *θ* of the policy *π* (cf. Eq 2).

Though, as the expected value of a certain state-action pair varies according to the policy itself, updating a new policy from samples acquired from an old policy may cause inaccurate predictions, as the expected value of an action-state pair may be wrong with respect to the new policy. PPO’s efficiency is due to the use of a trust region within which a policy update is deemed reasonable, which is an idea originally proposed in the TRPO algorithm [21]. PPO extends this idea by integrating a Kullback-Leibler divergence term to measure the breadth of an update directly within the objective function (a technical description is available elsewhere [8, 22]).

As we are dealing with episodes and do not want to encourage the focal agent to act in the least amount of time steps as possible, the discount factor is set to *γ* = 1.0, as recommended by Sutton et al. [10, p.68]. The PPO hyper-parameters used are reported in Table 1.

**Table 1:** Parameters used for the PPO algorithm. See Supplementary Materials for an extensive analysis of parameter sensitivity).

Parameters	Values
Learning rate	0.005
Optimiser Algorithm	SGD
Number of optimisation epochs	10
Minibatch size	128
Batch size	4000
Discount factor *γ*	1.0
Search space PPO-MLP (*θ*_*MLP*_)	$R^{33}$
Search space PPO-DEEP (*θ*_*DEEP*_)	$R^{133894}$

The investment and choice modules are both represented as Artificial Neural Networks (ANN). A module is composed of both a decision network and a Value function, as PPO runs as an actor-critic algorithm. The Value function network has the same layout as the decision network, but only output the (continuous) value of the state.

The decision network for the investment module is a simple neural network with one single input set to 1.0, no hidden layer and two outputs: the investment mean *m* and standard deviation *σ*. The investment *x*_•_ is picked along the distribution $N(m,\sigma^{2})$ and clipped between 0 and 15. The continuous stochastic action selection is essential to the PPO search algorithm.

The decision network for the choice module is a multilayer perceptron with two input neurons and two output neurons (for accepting or refusing cooperation). The output neurons use a linear activation function, and a softmax probabilistic choice is done to choose which action to make (accept or decline). Hidden units use an hyperbolic tangent activation function. A bias node is used, that projects on both the hidden layer(s) and output neurons. The Value Function estimator use the same architecture as the choice neural networks, with only one output.

In Section 4, two different architectures are evaluated, which we refer to as **PPO-MLP** and **PPO-DEEP**. While both use the decision network for the investment module described before, they differ with respect to the architecture used for the choice module. PPO-MLP implements a single hidden layer with 3 neurons, and PPO-DEEP implements a deep architecture with two hidden layers, each with 256 neurons. While PPO-DEEP may seem overpowered at first sight, over-parametrization has been shown to be very effective in deep learning as multiple gradients can be followed in wide neural networks [23–25].

All parameter values and module architecture result from an extensive search (summarised in the Supplementary Materials). In particular, a grid search was performed to select the best values for each parameters, including the learning rate (*lr*). The number of Simple Gradient Descent iterations, the batch size and the mini-batch size had little impact on neither performance nor convergence. In addition, we performed additional experiments to evaluate the impact of using (1) a discount factor *γ* < 1.0 (i.e. 0.9, 0.99 and 0.999) and (2) PPO without actor-critic. None of these settings provided better (or even comparable) results to those obtained with the parameters used in Table 1.

### 3.2 Covariance matrix adaptation evolution strategy

The Covariance Matrix Adaptation Evolution Strategy (CMAES) is an optimisation algorithm that does black box optimisation and is derivative-free [9]. The goal of CMAES is to find θ* that maximizes (or minimizes) a continuous function *f*. CMAES does not require the function to be convex or differentiable, and relies on stochastic sampling around the current estimate of the solution. CMAES creates a population of size λ using a multivariate Gaussian distribution. Each individual of the population is evaluated and CMAES then updates its distribution estimation based on the average of the sampled agents weighted by their evaluation rank. Furthermore, the covariance matrix of the multivariate Gaussian distribution is continuously updated so that the distribution is biased toward the most promising direction. A comprehensive introduction to the algorithmic foundations of CMAES can be found elsewhere [26].

The investment module is represented as a single real value (the investment), which is clipped between 0 and 15 when used. The partner choice module is a neural network with 2 inputs, one hidden layer with three neurons and two neurons on the output layer used to compute the probability to *accept* or *refuse* cooperation. A softmax probabilistic choice is made to choose which action to make. A bias node is also used, neurons from the hidden layer use an hyperbolic tangent activation function, and the output units use a linear activation function. There are 17 neural network weights.

The parameters for both modules are compiled into a single vector of real values. To make the search space similar to that of PPO, dummy parameters are added to the vector (i.e. values which can be modified by the algorithm, but with no impact on the outcome) to reach a total number of 34 real values (i.e. $\Theta \in R^{34}$).

Table 2 summarizes the parameters used for the CMAES algorithm.

**Table 2:** Parameters for the CMAES algorithm.

Parameter	Value
Population size	14
Number of episode per evaluation	1
*σ*_*init*_	1.0
Search space (*θ*_*CMAES*_)	$R^{34}$

As CMAES is mostly parameter-free, there were no need to perform extensive preliminary search. We used the default values and let the algorithm automatically set its internal parameters (automatic parameter tuning in CMAES has been considered others [26, 27]). We choose *σ*_*init*_ = 1.0 for the initial standard deviation and a vector of zeros as initial guess. The population size λ is the default population size in the python CMAES implementation [28], i.e. λ = 4 + ⌊3 × ln(*N*)⌋ = 14 with *N* the number of dimensions in the model. Once the λ candidate solutions are evaluated, a new population is generated according to their performance. A new population is generated every 14 episodes, and so forth until the evaluation budget is consumed.

A candidate solution for the focal agent is evaluated on one episode only, which length may vary depending on when the focal agent and its partner both accepts to cooperate (maximal duration defined in Eq 4).

## 4 Results

The environment, the models and the learning algorithms are implemented with ray [29], rllib [30] and pytorch [31]. We use the cma [32] package in python for the CMAES implementation. Source code is available at https://github.com/PaulEcoffet/RLCoopExp/releases/tag/v1.1.

For a given value of probability of rare significant events *p*, we performed 24 independent runs for each algorithm. A run lasts 200000 episodes. The maximum duration of an episode is fixed as described in Section 2.1 so the expected number of significant events remains identical independently from the *actual* rarity throughout one episode (cf. Eq 4). In practical, an episode lasts *at most* 100 (resp. 200, 500, 1000) iterations for *p* = 1.0 (resp. 0.5, 0.2, 0.1). While *p* = 1 implies that all interactions between the focal agent and its partners are significant, setting *p* = 0.1 implies that the focal agent will experience *very few* significant events where its actual partner may be ready to cooperate. It can be expected (and will be shown in the following) that the level of cooperation depends on the value of *p* and the learning algorithm used, especially with lower value of *p*.

Performance of the current policy is plotted every 4000 iterations, which corresponds to the batch size used by both PPO instances for learning. As episodes last significantly shorter than 4000 iterations this means the policy’s performance is averaged. For CMAES, we extract the best policy of the current generation and re-evaluate it 10 times (i.e. for 10 episodes) to get a similarly averaged performance. Results are shown on figures with a data point every 1000 episodes.

### 4.1 Learning when all events are significant

**Fig 1:**
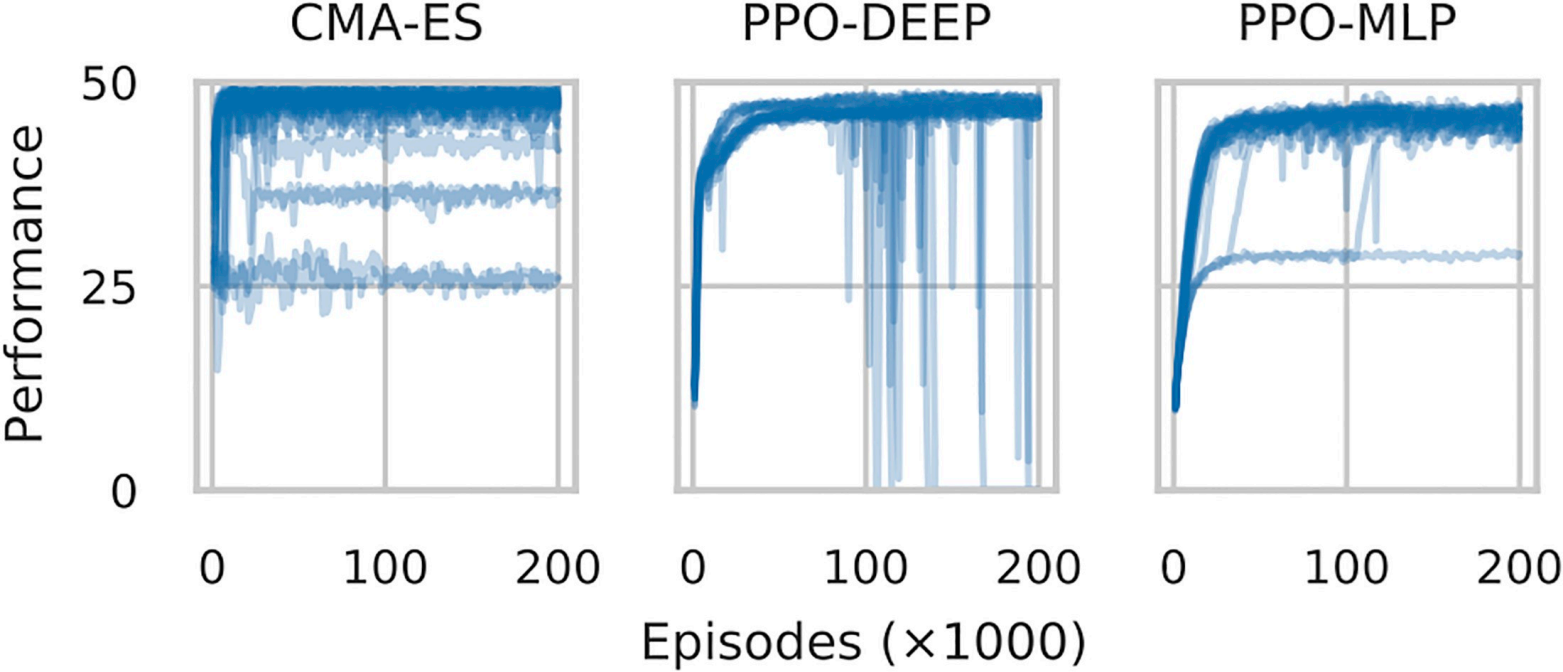
Performance of the best policy throughout learning with CMAES (top), PPO-DEEP (center) and PPO-MLP (bottom), with 24 independent runs per method, for 200 * 10^3^ episodes. There are 20/24 runs that produced a policy where performance above 40 with CMAES, 20/24 for PPO-DEEP and 23/24 for PPO-MLP. Note that PPO-DEEP produces 24/24 runs with performance above 40 around episode 80 * 10^3^, with performance occasionally degrading and immediately recovering for some runs afterwards due to the learning step size (see Annex for further analysis).

Fig 1 shows the performance throughout learning for CMAES, PPO-DEEP and PPO-MLP when *p* = 1.0 (i.e. the focal agent faces only cooperative partners). Each Figure shows 24 curves corresponding the 24 independent runs. Both PPO versions and CMAES are shown to learn near optimal policies (*performance* → 50) in almost all runs. CMAES is the fastest to converge, and PPO-DEEP (despite the huge number of dimensions) is faster than PPO-MLP. On the other hand, CMAES offers less robustness as 20 (out of 24) runs with CMAES reach a performance above 40, to be compared to 23 (out of 24) runs with PPO-MLP and 24 runs with PPO-DEEP.

**Fig 2:**
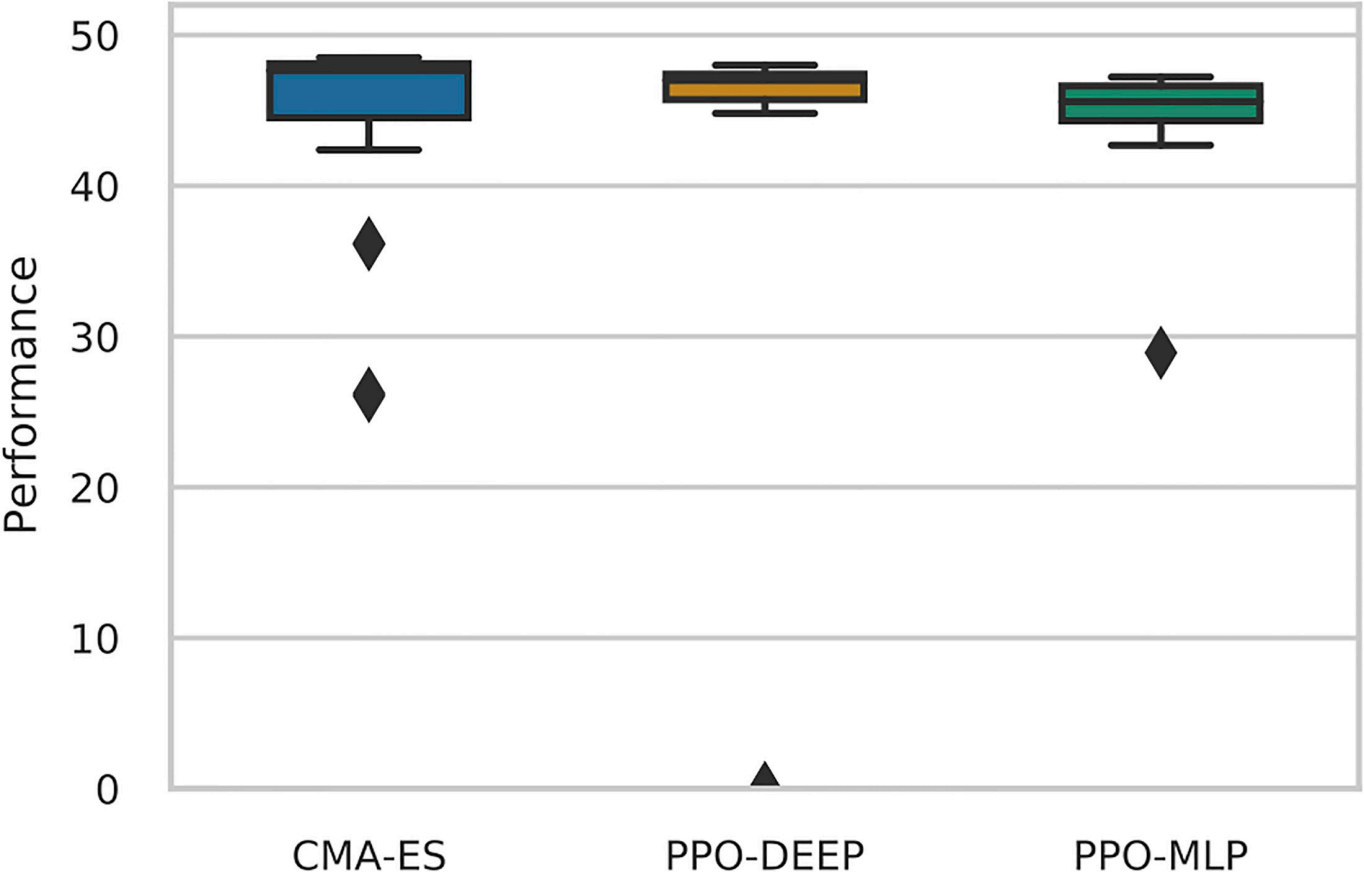
Performance of the best policies from CMAES, PPO-DEEP and PPO-MLP with *p* = 1.0 after re-evaluating policies for 1000 episodes without learning. Two-tailed Mann-Whitney U-test, *n* = 24, gives *p*-value = 0.12 (CMAES vs. PPO-DEEP), *p*-value = 0.019 (CMAES vs. PPO-MLP), *p*-value = 0.018 (PPO-DEEP vs. PPO-MLP). Median values and Median Absolute differences are: CMAES (median = 47.64, MAD = 4.72) is only slightly more than that of PPO-DEEP (median = 46.99, MAD = 13.04) and PPO-MLP (median = 45.58, MAD = 1.86).

In order to better compare the quality of the policies learned by each algorithm, the best policy from the end of each run is selected and re-evaluated for 1000 extra episodes without learning. Results are shown in Fig 2 with all three methods faring similar performance. The median value for CMAES (47.64) is only slightly more than that of PPO-DEEP (46.99) and PPO-MLP (45.58).

Therefore, we conclude that all three algorithms provide excellent and comparable results when only significant events are experienced (*p* = 1.0).

### 4.2 Learning when significant events are rare

**Fig 3:**
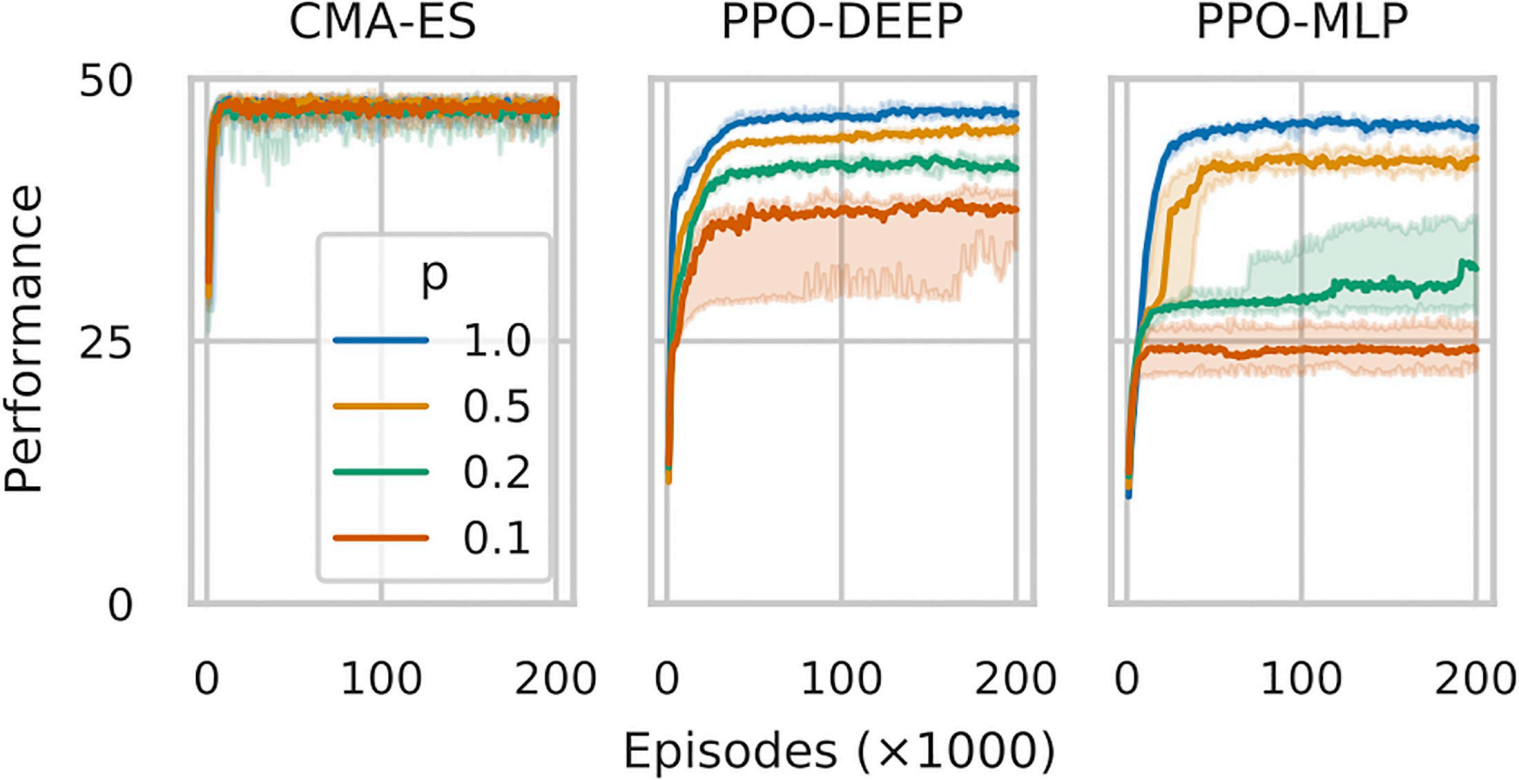
Performance of the best policies (median and 95% confidence interval) throughout learning with CMAES, PPO-DEEP and PPO-MLP for the 3 conditions with rare significant events (*p* ∈ {0.1, 0.2, 0.5}) and 1 control condition (*p* = 1.0, same data as shown in Fig 1), for the first 75 * 10^3^ episodes (out of 200 * 10^3^).

Fig 3 show the performance of the agent throughout its learning with both PPO algorithms and the CMAES algorithm for different conditions of rare significant events (*p* ∈ {0.1, 0.2, 0.5}), as well as with the control condition when all events are significant (*p* = 1.0, taken from the previous Section). Each figure shows the mean performance of 24 independent runs per conditions, compiling each setup by tracing the median performance and 95% confidence interval from the 24 runs.

CMAES is only marginally impacted when significant events become rarer (i.e. *p* < 1.0), with all setups showing convergence towards a similar performance value close to the optimal (above 40). While PPO-DEEP fares better than PPO-MLP for *p* < 1.0, both are largely affected. In the extreme case where *p* = 0.1, the average performance of 35.7 ± 5.2 for PPO-DEEP and 24.9 ± 4.2 of PPO-MLP, to be compared to 46.2 ± 3.2 for CMAES.

**Fig 4:**
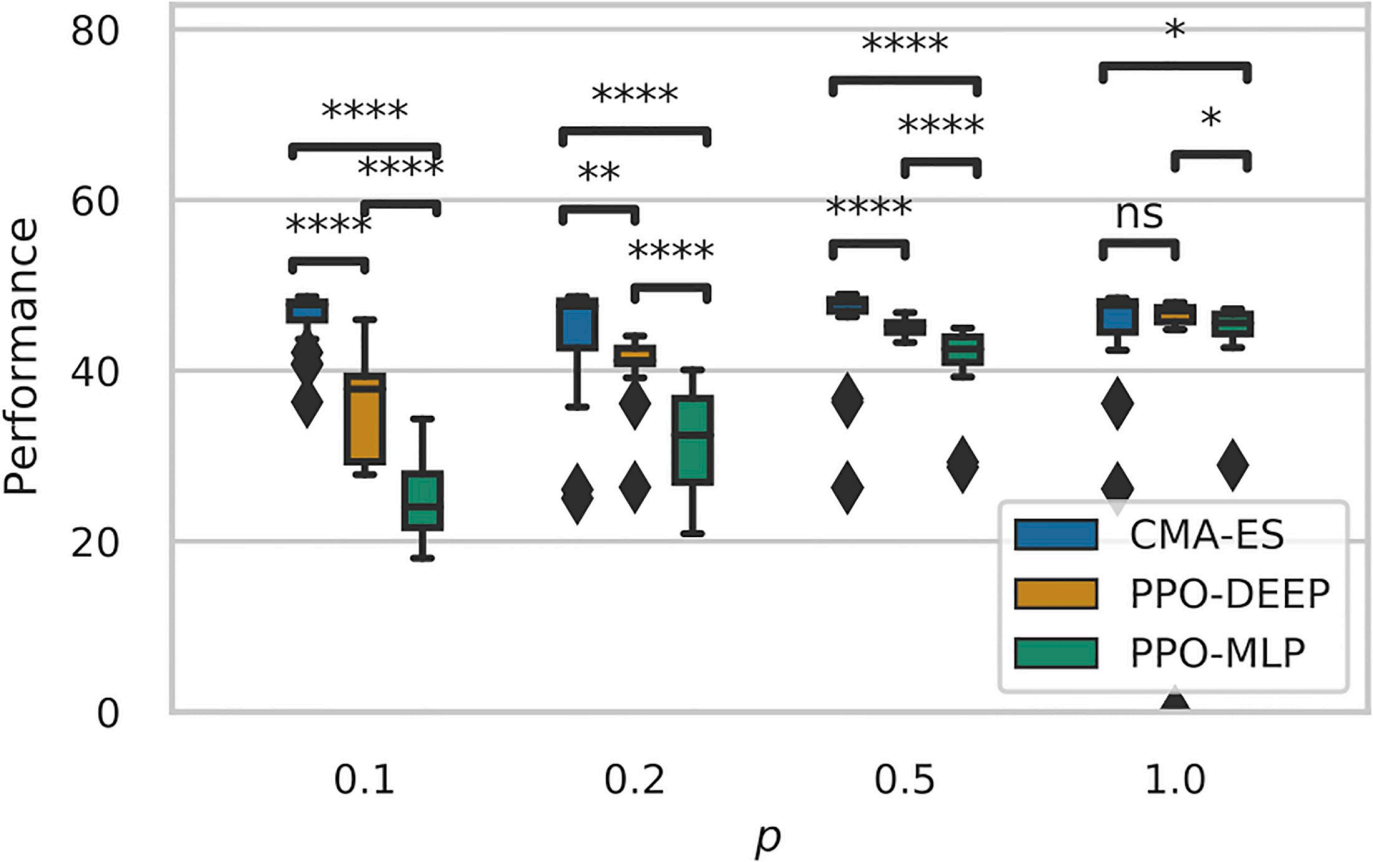
Performance of the best policies (medians and quartiles) from CMAES, PPO-DEEP and PPO-MLP with *p* ∈ {0.1, 0.2, 0.5, 1.0} after re-evaluating policies for 1000 episodes without learning. Two-tailed Mann-Whitney U-test, *n* = 24 marked as: * for *p*-value < 0.05, ** for *p*-value < 0.01, *** for *p*-value < 0.001 and **** for *p*-value < 0.0001.

Fig 4 shows the results for the additional analysis where the best policy from each run for each condition *p* ∈ {0.1, 0.2, 0.5, 1.0} is selected and re-evaluated for 1000 extra episodes without learning and with the condition *p* = 1.0 (i.e. only significant events matter here). Results confirm that the difference in the performance of policies obtained with CMAES compared to either versions of PPO widens as significant events become rarer (*p* < 1.0) with both PPO-MLP and PPO-DEEP faring significantly worse than CMAES (*p*-value <0.0001, Mann-Whitney U-test).

### 4.3 Analysing the best policies for partner choice

In order to better understand why policies’ performance differ among learning algorithms and conditions, the agent’s policy obtained at the end of each run is extracted and analysed (i.e. 24 policies per algorithm per condition).

**Fig 5:**
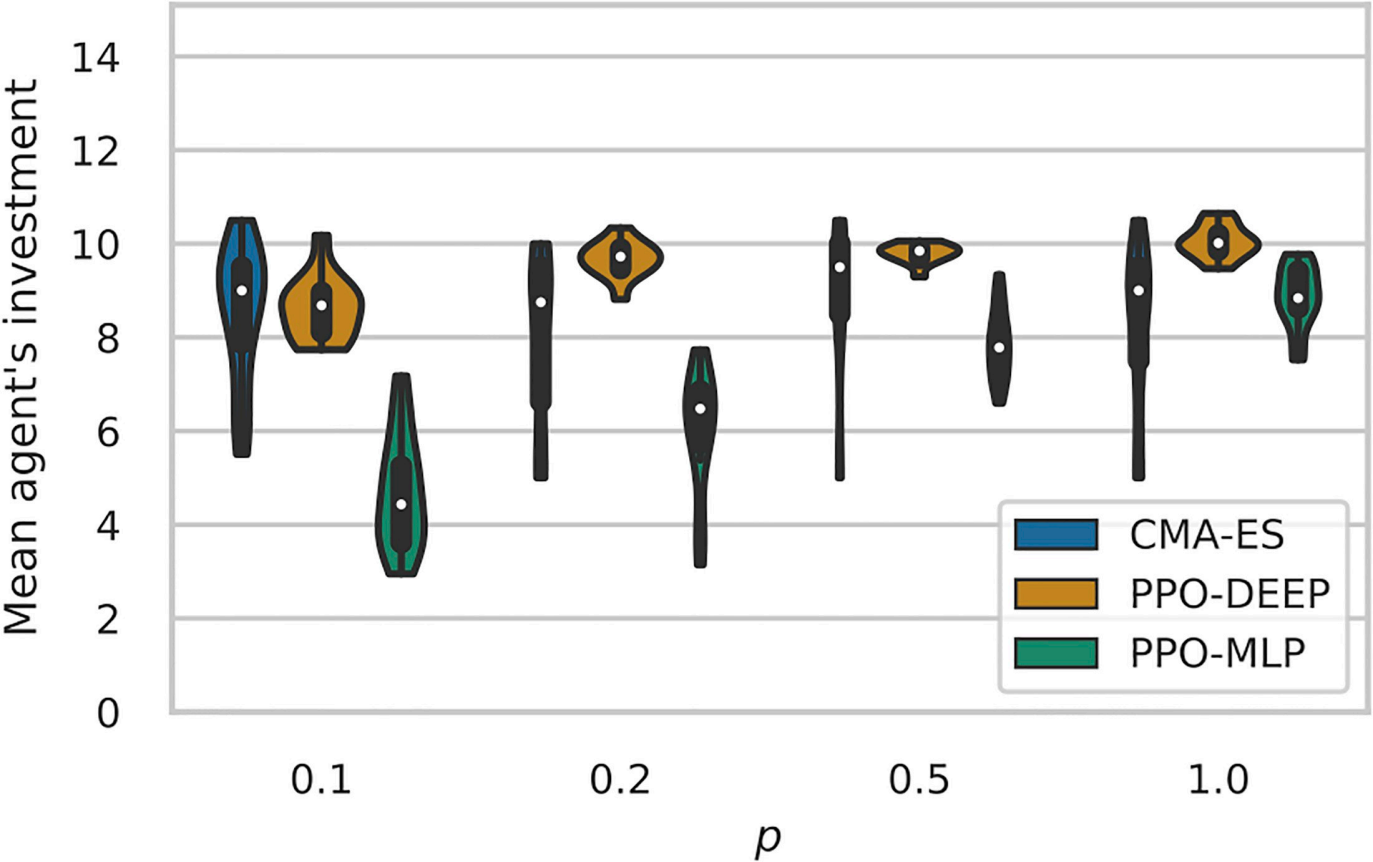
Investment value of the focal agent given by the Investment Module for the best learned policies with CMAES (blue), PPO-DEEP (orange) and PPO-MLP (green) algorithms, for each condition *p*. Each violin graph represents the results of the outcome of the 24 best policies for a given algorithm and condition after being re-evaluate for 1000 episodes without learning.

Fig 5 illustrates the outcome of the Investment Module (*x*_•_), i.e. the investment value offered by the focal agent when faced with a potential partner. It is obtained by measuring the investment value of the focal agent from 1000 episodes with *p* = 1.0 and without learning.

Policies learned with CMAES play close to *x*_*c*_ = 10, which is the optimal play for the payoff function (Section 2.2), whatever the frequency of significant events. As expected, this is different for policies learned with PPO, as the outcome values of the Investment Module are significantly lower when the frequency of significant events decreases (*p* < 1.0).

**Fig 6:**
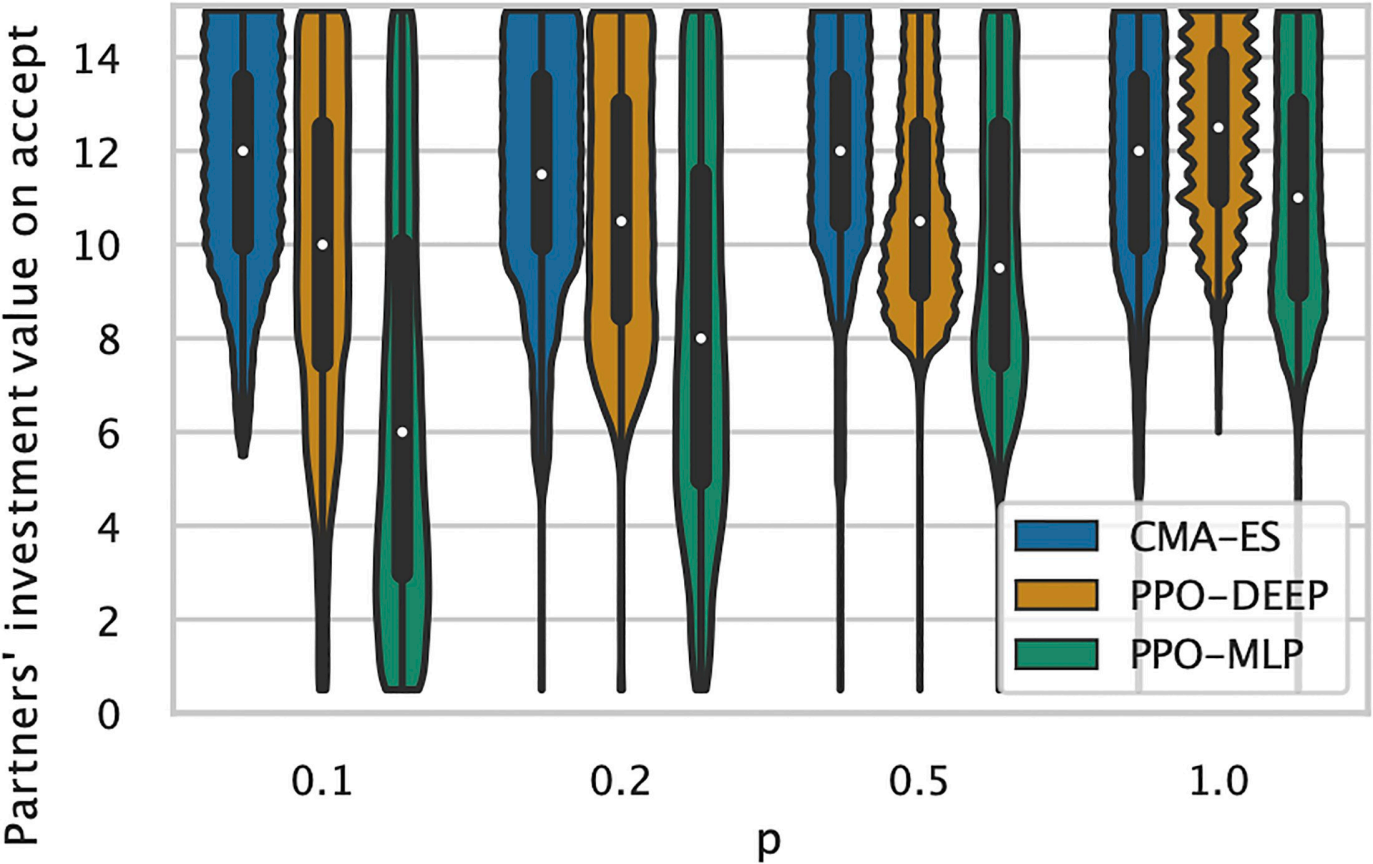
Decision to accept to cooperate taken by the focal agent, when facing a cooperative partner with a particular investment value. Results for CMAES (blue), PPO-DEEP (orange) and PPO-MLP (green) are shown as violin graph. X-axis: algorithms and conditions, Y-axis: partner’s investment value for which the focal agent accept to cooperate.

Fig 6 illustrates the investment values played by cooperative partners, when the focal agent accepts to cooperate (whether or not cooperation will actually take place, as it also depends on the partner’s acceptance). In other words, it represents how demanding is the focal agent with respects to its partners’ intention to invest in cooperation. The probability to accept cooperation is computed for the policies of each run. Each policy is presented with all 31 possible cooperative partners, 100 times each, to estimate the focal agent strategy. While CMAES produced consistent policies that follow quasi-identical strategies for all conditions (ie. accepting partners that invest close to the optimal *x*_*c*_ = 10 or above), this is not the case for PPO policies which are less demanding for lower value of *p*, with many of the policies learned by PPO-MLP with condition *p* = 0.1 actually accepting *any* partners). PPO-DEEP policies fare better than PPO-MLP policies, but still worse than policies learned with CMAES when significant events are rarer.

**Fig 7:**
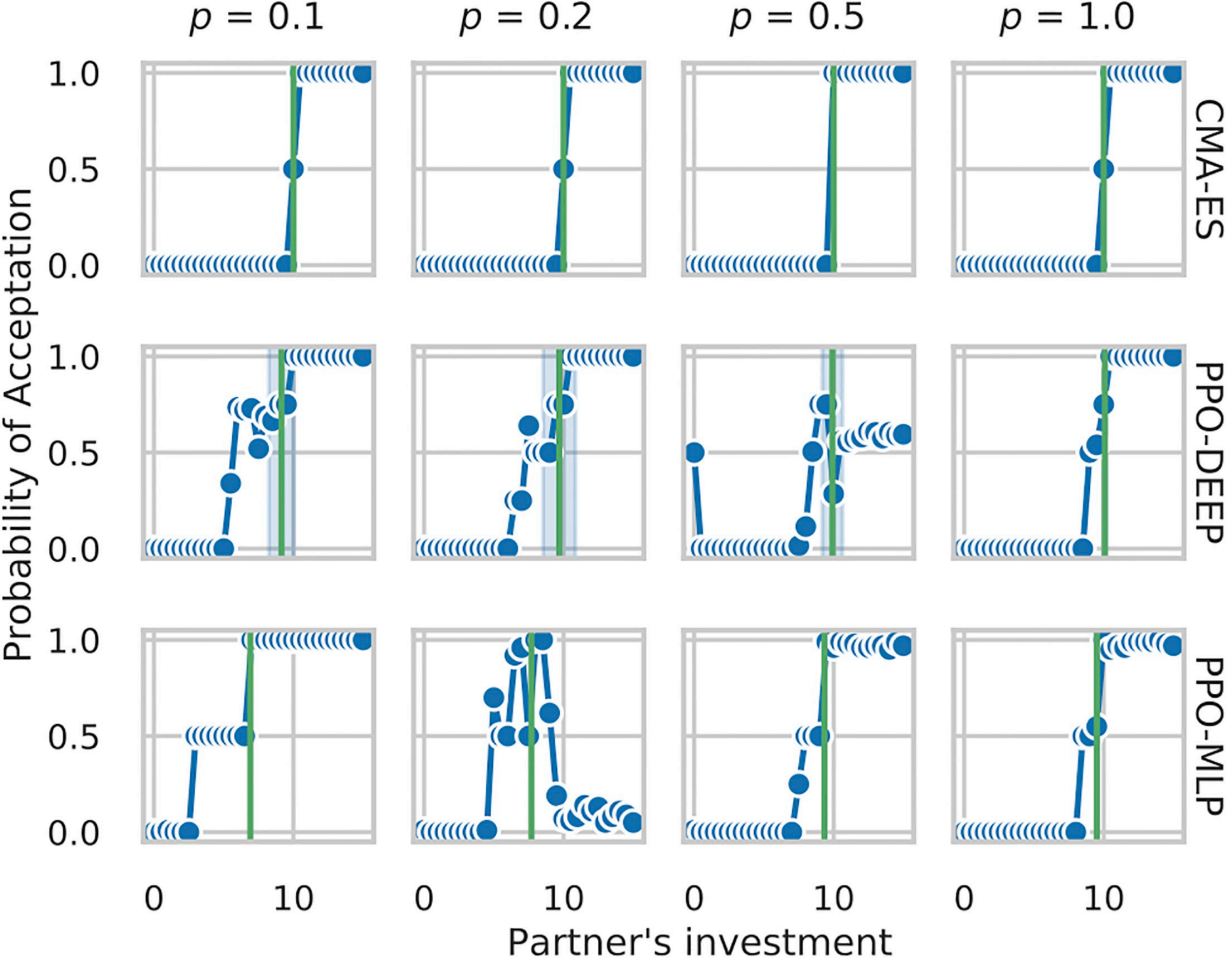
Analysis of the Partner Choice module for all conditions (by columns: *p* ∈ {0.1, 0.2, 0.5, 1.0}) and all algorithms (top: CMAES, center: PPO-DEEP, bottom: PPO-MLP). For each setup, only the best policy is shown. Each graph plots the probability to accept cooperation for the focal agent following the best policy (y-axis) depending on its partner’s proposed investment (x-axis). Data are computed by presenting each of the 31 possible cooperative partners to the focal agent for 100 iterations as policies are stochastic. The green vertical line represent the mean investment of the focal agent.

Fig 7 takes a detailed look at the results shown in Fig 6. It shows the strategy profile for partner choice by the *best* policy obtained with each algorithm in each condition. Focal agents obtained with CMAES follow an efficient and clear-cut strategy: they play the optimal investment value (*x*_•_ = *x*_*c*_ = 10, green vertical line) and accept partners only when those play a similar or better value ($x_{i}^{+}\geq 10$, blue line). Policies obtained with PPO-DEEP and PPO-MLP either follow *roughly* the same profile with a more stochastic behaviour (PPO-MLP policies for *p* = 1.0 and 0.5, PPO-DEEP policies for *p* = 1.0, 0.2 and 0.1) or display a selective strategy, choosing partners only when they play close to the optimal investment value $x_{i}^{+}\approx x_{c}$. Only PPO-MLP produced policies which are clearly sub-optimal for *p* = 0.2 and *p* = 0.1, with a mean investment below the optimal investment value *x*_•_ < *x*_*c*_.

## 5 Concluding remarks

In this article, we focused on an on-policy reinforcement learning problem of an autonomous agent that needs to maximize its gain when interacting with other agents, with whom our agent may or may not decide to cooperate. The peculiarity of this problem is to present a (very) small number of significant events during which the agent can obtain only one single positive reward. The challenge is therefore to learn how to best choose a partner, by making a compromise between the chances of finding a better partner, and the cost of an interaction.

We studied the dynamics of two reinforcement learning methods: a gradient policy search algorithm and a direct policy search algorithm with an evolution strategy. Both algorithms succeeded in learning policies that make an optimal use of partner choice when interaction opportunities are frequent. However, the two algorithms differ fundamentally when interaction opportunities are rare. The direct policy search algorithm shows total robustness, while the gradient policy search algorithm collapses, resulting in sub-optimal policies.

The robustness of the direct policy search method can be expected as the sequential and temporal aspects of the task is lost within one evaluation. As long as the evaluation time is long enough to sample the whole population of relevant partners, there is no cost nor change in the algorithm dynamics to deal with a situation where significant events are lost within a longer sequence, but still of the same number. Such independence to action frequency and delayed rewards have actually been observed elsewhere, though for different problems (e.g.: robotic control problem [33]). This is of course different for the gradient policy search method, where increased rarity means that many learning steps will be performed with zero-reward, resulting in poor gradient information most of the time. Not only this slows down learning, even with a similar number of iterations, but it also prevents learning from converging towards a truly optimal partner choice strategy. This remains true even when a large search space is considered, in which over-parametrization in deep neural networks help gradient search [23, 24].

The broader motivation behind this work is to identify reinforcement learning problems for which evolutionary algorithms as a direct policy search method offer a competitive advantage over gradient policy research methods (see also works from other authors [33–39]). The take-home message that emerges from this paper is that one of these problems occurs when important events are rare, for which direct policy search shows an invariance to rarity.

As a final remark, it may be tempting to relate the problem of rare significant events with that of sparse rewards, which has gain a lot of attention recently [40–42]. However, they differ fundamentally as significant events may be rare, but *eventually* occur. This is not the case with sparse rewards, which occurrences are conditioned by the policy itself (e.g. a robotic arm must be within the length of a target to trigger a reward) and may *never* be obtained. We also argue that problems where significant events are rare rather than sparse may be more numerous than expected: a complex environment offers multiple learning opportunities, as long as one is able to seize them as they arise.

## Supporting information

S1 Appendix (PDF)

S1 Annex Notation. (PDF)
