# Supplementary material for: Policy search with rare significant events: Choosing the right partner to cooperate with
Source: PLoS One. 2022 Apr 26;17(4):e0266841. doi: 10.1371/journal.pone.0266841 (PMC9041856; doi:10.1371/journal.pone.0266841)
Supplement: S1 Appendix — (PDF) [file pone.0266841.s001.pdf]

arXiv:submit/3645823 [cs.LG] 11 Mar 2021

Policy Search with Rare Significant Events: Choosing the Right Partner to Cooperate with

March 11, 2021

A Supplementary materials

A.1 Detail analysis of the agents' reward

We plot the performance per run of each conditions in Fig. 1. As p gets lower, the performance reached by a run gets slightly lower. Furthermore, for $p \in \{1, 0.5\}$, agent's reward converges to two different values: one optimal equilibrium above 40 and a second sub-optimal equilibrium at around 30. There are two equilibria that the algorithm reaches. The transition from the sub-optimal equilibrium to the optimal equilibrium is quick, but occurs less and less often as p gets smaller.

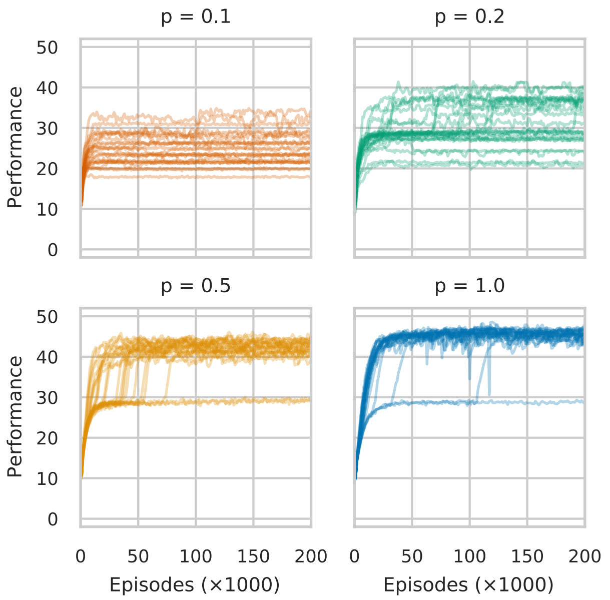

Figure 1: Split view of the different performances with PPO-MLP. For $p = 1.0$, in most of the simulations, the agent reaches a performance close to the optimal performance at the end of its learning. In a few simulations, the agent gets stuck at a plateau value. If the agent overcome this plateau value, it reaches quickly the close-to-optimal performance. For $p = 0.5$, in most of the simulation the agent's performance gets close to the optimal reward value. The plateau is reached more often than in the $p = 1.0$ condition. The plateau's equilibrium is even stronger for $p = 0.2$ and $p = 0.1$. In almost no simulation to no simulation at all the agent's performance escape from the plateau equilibrium to reach the close-to-optimum equilibrium.

The detailed analysis of the performances through the episodes of the CMAES condition shows the same pattern as the PPO-MLP conditions for $p = 1.0$. For smaller values of p, CMA-ES agents gets still close to the optimal performance, whereas PPO-MLP agents' performances plunger.

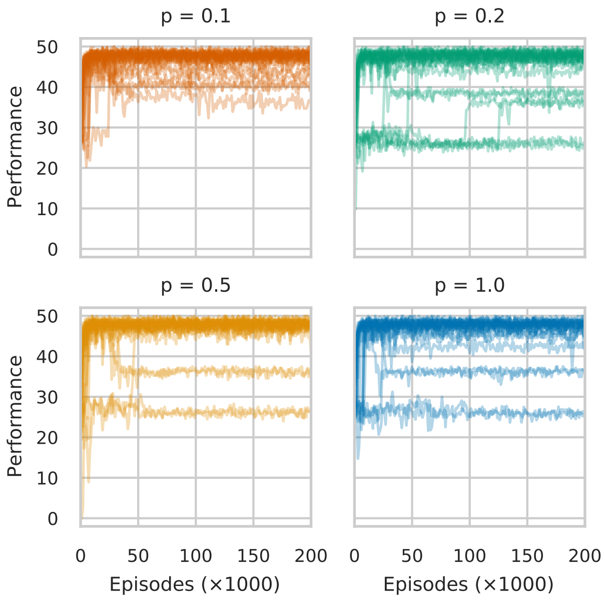

Figure 2: Split view of the different performances of CMA-ES for different values of p. Regardless of the value p, in most of the simulations the agent gets a performance close to the optimal performance at the end of the learning. Like the PPO-MLP condition, some simulations reach a sub-optimal equilibrium around 30. Few of them manage to get out of this equilibrium.

The detailed analysis of the performance through the episodes of the PPO-DEEP condition shows the same pattern as the PPO-MLP conditions. Though, the performance loss as p gets lower is far less strong.

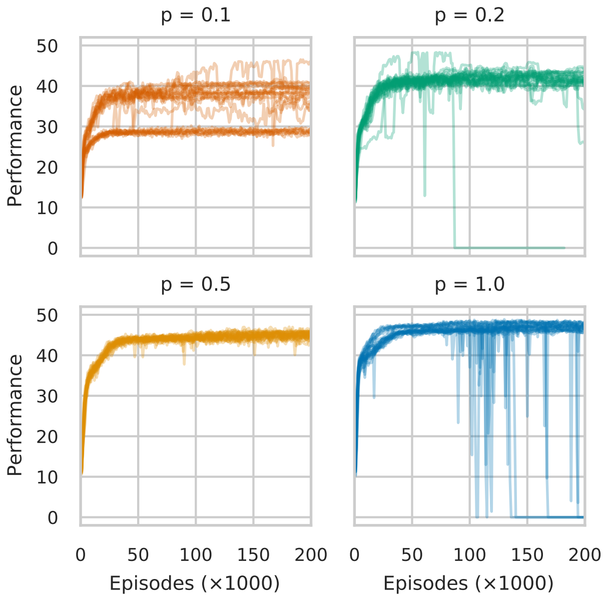

Figure 3: Split view of the different performances of PPO-DEEP for different values of p. Performance varies with p, the higher p, the better the performance. The loss in performance as p gets lower is less strong than with PPO-MLP. Performance is highly unstable after 100,000 generations for $p = 1.0$.

A.2 Re-evaluation performance statistical score

We perform two-tailed Mann-Whitney's U-tests to compare the distributions of the performance for the PPO-MLP, PPO-DEEP and CMA-ES agent for each probability p of meeting a x^+ partner. The table of the median performance of each learning algorithm for each p is reported in table 1 and the U-statistics and p-values of the tests are reported in table 2.

p	Algorithm	Median	MAD	Mean	Std
0.10	CMA-ES	47.72	2.45	46.19	3.21
	PPO-DEEP	37.83	4.43	35.73	5.16
	PPO-MLP	24.04	3.43	24.92	4.23
0.20	CMA-ES	47.56	5.32	44.03	6.98
	PPO-DEEP	41.18	1.90	40.76	3.57
	PPO-MLP	32.44	5.20	31.60	5.93
0.50	CMA-ES	48.00	4.59	45.17	6.67
	PPO-DEEP	45.23	0.70	45.06	0.89
	PPO-MLP	42.52	2.62	41.49	4.17
1.00	CMA-ES	47.64	4.72	44.42	6.62
	PPO-DEEP	46.99	13.04	39.12	17.89
	PPO-MLP	45.58	1.86	44.78	3.62

Table 1: Median of the re-evaluations, 24 runs per condition

p	test	$p - value$	U-statistic
0.1	PPO-MLP vs PPO-DEEP	1.2e-07	31
	PPO-MLP vs CMA-ES	3.1e-09	0
	PPO-DEEP vs CMA-ES	3.9e-08	21
0.2	PPO-MLP vs PPO-DEEP	2.5e-07	33
	PPO-MLP vs CMA-ES	3e-06	61
	PPO-DEEP vs CMA-ES	0.0023	132
0.5	PPO-MLP vs PPO-DEEP	6.4e-07	46
	PPO-MLP vs CMA-ES	5.5e-05	92
	PPO-DEEP vs CMA-ES	9.3e-05	98
1.0	PPO-MLP vs PPO-DEEP	0.018	173
	PPO-MLP vs CMA-ES	0.019	174
	PPO-DEEP vs CMA-ES	0.12	212

Table 2: Statistical results of the two-tailed Mann-Whitney U-test comparing the performance of the agents using PPO-MLP, PPO-DEEP and CMAES in the re-evaluation setup. $n = 24$ for each condition and algorithm.

A.3 Timing

We measure the execution time (wall time) for both PPO-MLP and CMAES on a single CPU. To do so, we take the best agent out of the 24 simulations for each algorithm and each condition, and restart the learning from its state for 15 minutes. We then divide the total time taken by the algorithm by the number of episode time step the algorithm fulfilled.

CMA-ES overhead is constant, as CMA-ES updates are more frequent per episode time steps as p increases. Indeed, the shorter the episodes are, the more often CMA-ES updates triggers. PPO-MLP and PPO-DEEP updates are constant with respect to the episode length, as they always update when 4000

episode time steps are completed. The PPO-MLP execution time compare to the CMA-ES execution time is explain by the RLlib overhead as well as its learning computational cost. The PPO-DEEP execution time is greater than PPO-MLP execution time. It is explained both by the larger network that requires a more computationally intensive evaluation, as well as the gradient descent which involve massively more weights.

The total time divided by the number of iteration is reported in Figure 4 as well as in Table 4. Speed ratio are shown in Table 3

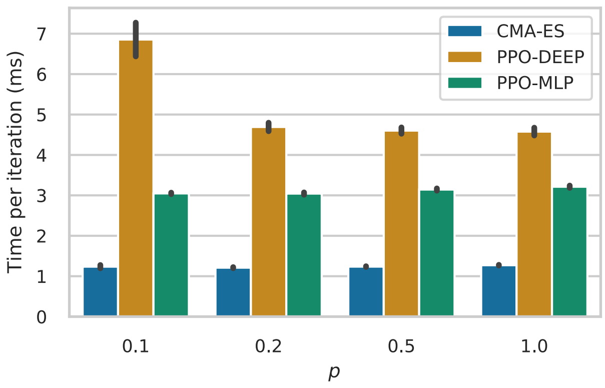

Figure 4: Average time per episode time steps (Learning included) for CMAES, PPO-DEEP and PPO-MLP for the different conditions p.

Algorithm	p	Time per step (ms) Mean ± std
CMA-ES	0.10	1.24 ± 0.04
	0.20	1.21 ± 0.01
	0.50	1.24 ± 0.01
	1.00	1.27 ± 0.01
PPO-DEEP	0.10	6.86 ± 0.43
	0.20	4.69 ± 0.11
	0.50	4.60 ± 0.08
	1.00	4.58 ± 0.10
PPO-MLP	0.10	3.05 ± 0.02
	0.20	3.04 ± 0.03
	0.50	3.14 ± 0.03
	1.00	3.21 ± 0.03

Speed ratio col/row	CMA-ES	PPO-DEEP	PPO-MLP
CMA-ES	1.00	4.18	2.51
PPO-DEEP	0.24	1.00	0.60
PPO-MLP	0.40	1.66	1.00

Table 3: Speed ratio between algorithms

Table 4: Table of computational wall time per environmental time step for CMA-ES, PPO-DEEP and PPO-MLP with one single core. The average time per environmental step includes the time needed by the learning algorithm to update the policy.

A.4 Influence of the discount factor

We tested different value of the discount factor γ for PPO-MLP. These results are reported in Figure 5. Using a $\gamma < 1$ has a detrimental impact on the agent's performance regardless of the value of p. The agent is always stuck in the suboptimal plateau of a performance of 25. With $\gamma = 0.999$ and $p = 1.0$, the agent gets a performance close to the performance of the PPO-MLP agents with $\gamma = 1$.

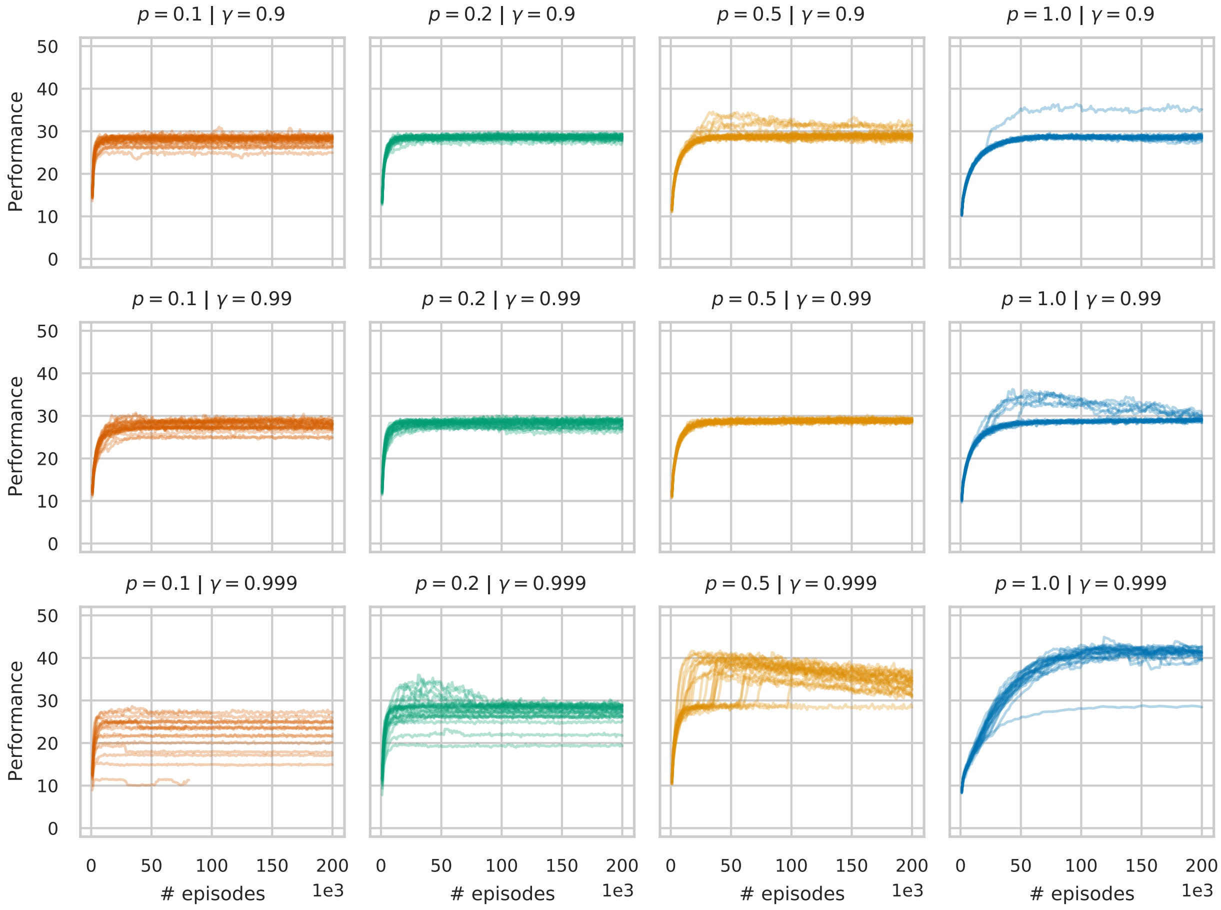

Figure 5: Performance of the PPO-MLP agent for different values of discount factor $\gamma \in \{0.9, 0.99, 0.999\}$. Low γ has a negative impact on performance compared to $\gamma = 1$. The best results are obtained for $\gamma = 1$.

A.5 Influence of the absence of critic

The absence of a critic does not have any positive influence on the performance of the PPO-MLP agent. The performance is much nosier and below the performance of the PPO-MLP agent with a critic and GAE.

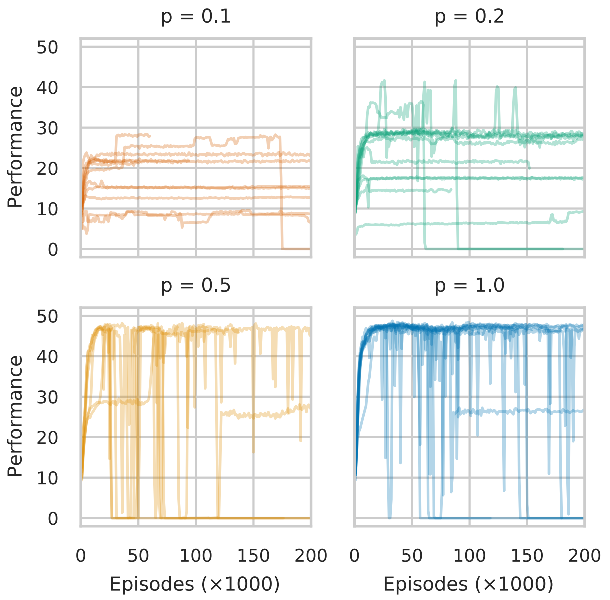

Figure 6: The absence of critic does not improve PPO performance.

A.6 PPO Loss Function

Our PPO implementation optimize θ by maximising the function $LL^{CLIP+KLPEN}(\theta)$ given in Equation 1.

$$\begin{aligned} L^{CLIP+KLPEN}(\theta) = & \hat{\mathbb{E}}_t[\min\left(r_t(\theta)\hat{A}_t, \operatorname{clip}\left(r_t(\theta), 1-\epsilon, 1+\epsilon\right)\hat{A}_t\right) \\ & - \beta \mathrm{KL}\left[\pi_{\theta_{\text{old}}}\left(\cdot \mid s_t\right), \pi_\theta\left(\cdot \mid s_t\right)\right]] \end{aligned} \tag{1}$$

with $r_t(\theta) = \frac{\pi_\theta(a_t|s_t)}{\pi_{\theta_{\text{old}}}(a_t|s_t)}$, $\hat{A}_t$ the estimator of the advantage at time step t. β varies according to the KL target d_{targ} (here 0.01). $\beta = 0.2$ at the beginning of the simulation and adjusts at each time step according to the following rule, be $d = \mathbb{E}_t\left[\mathrm{KL}\left[\pi_{\theta_{\text{old}}}\left(\cdot \mid s_t\right), \pi_\theta\left(\cdot \mid s_t\right)\right]\right]$:

$$\begin{cases} \beta \leftarrow \beta \times 1.5 & \text{if } d > 2 \times d_{targ} \\ \beta \leftarrow \beta / 2 & \text{if } d < d_{targ}/2 \end{cases} \tag{2}$$

All the PPO-MLP and PPO-DEEP hyperparameters are available in Table 5.

**Parameters**	**Values**
Learning rate PPO-MLP	0.005
Learning rate PPO-DEEP	0.001
Optimiser Algorithm	SGD
Number of optimisation epochs	10
Minibatch size	128
Batch size	4000
Kullback-Leibler coefficient β	0.2
Kullback-Leibler target d_{targ}	0.01
Discount factor γ	1.0
Search space PPO-MLP (θ_{MLP})	$\mathbb{R}^{33}$
Search space PPO-DEEP (θ_{DEEP})	$\mathbb{R}^{133894}$

Table 5: All parameters for the PPO algorithm
