# Supplementary material for: Policy search with rare significant events: Choosing the right partner to cooperate with
Source: PLoS One. 2022 Apr 26;17(4):e0266841. doi: 10.1371/journal.pone.0266841 (PMC9041856; doi:10.1371/journal.pone.0266841)
Supplement: S1 Annex — (PDF) [file pone.0266841.s002.pdf]

Annex: notations

Important notations are summarized in Table 3.

$x_\bullet$	the focal agent's investment value, with $x_\bullet \in [0, 15]$
$a_\bullet$	the focal agent's decision to actually cooperate, which depends on its own investment value $x_\bullet$ and that of its current partner. The agent accepts to cooperate only if $a_\bullet > 0$, with $a_\bullet \in \mathbb{R}$
x_I, x_J, ...	Agents who may (or may not) interact with the focal agent. Can also be named $x_{partner}$
x_i^+	investment value of an agent that may accept to cooperates ($x_i^+ \in X^+$), with $x_i^+ \in [0, 15]$.
a_i^+	a possibly cooperative agent's decision to pursue cooperation given its own investment value x_i^+ and that of the focal agent's investment value $x_\bullet$.
x_j^-	investment value of an agent that *always refuses* to cooperate ($x_j^- \in X^-$). x_j^- is always equal to 0.
a_j^-	a non-cooperative agent's decision to pursue cooperation, which is *always* negative.
p	probability that the focal agent encounters a cooperative agent at a given time step (e.g. $p = 1.0$ implies that all agents met may be willing to cooperate, the focal agent's investment value $x_\bullet$ is large enough).
T	maximum number of time steps for evaluating the focal agent
$J(\theta)$	return function (i.e. sum of reward $r(s, a)$ computed over one episode of maximum duration T
$r(s, a)$	reward at state s obtained with action a
$payoff(s, a)$	reward at state s obtained with action a, if the partner is a cooperative agent (i.e. x_i^+)
$P(x_\bullet, x_i^+)$	a function that computes the payoff between the focal agent (which invests $x_\bullet$) and a *cooperative* partner (which invests x_i^+), when both agree to cooperate.
x_d	the sub-optimal investment for the focal agent when partner choice is not possible.
x_c	the optimal investment for the focal agent, possible only with partner choice.

Table 3. Summary of the important notations used in the paper (see Section 2 for details).
